# Web-Based Short Video Intervention and Short Message Comparison of Repeat Blood Donation Behavior Based on an Extended Theory of Planned Behavior: Prospective Randomized Controlled Trial Study

**DOI:** 10.2196/37467

**Published:** 2022-12-23

**Authors:** Qiuyue Hu, Wei Hu, Wenjuan Han, Lingling Pan

**Affiliations:** 1 Blood Center of Zhejiang Province Hangzhou China

**Keywords:** extended theory of planned behavior, repeated blood donation intervention, randomized controlled trial, mobile phone

## Abstract

**Background:**

Although blood is an indispensable and important resource for clinical treatment, an imbalance between supply and demand may occur as the population ages and diversifies. Studies indicate that repeat blood donors are safe blood sources because of their voluntary blood donation education and frequent blood screening. However, the high rate of reduction in the number of first-time voluntary blood donors and low rate of repeated blood donation are common problems worldwide.

**Objective:**

This study aimed to evaluate the effect of an intervention in nonregular blood donors using web-based videos and SMS text messages, in which the former was guided by the extended theory of planned behavior, to discover effective intervention methods to improve repeat blood donation rates among nonregular blood donors.

**Methods:**

A total of 692 nonregular blood donors in Zhejiang province were randomly divided into intervention and control groups. The control group received regular, short reminder messages for a 6-month period, whereas the intervention group received web-based videos on the WeChat platform. The intervention group was guided by an extended theory of planned behavior, which included 9 factors: the respondents’ attitude, subjective behavioral norms, perceived behavioral control, the willingness to donate blood, outcome expectations, self-identity, blood donation–related anxiety, cognition of the blood donation environment, and previous blood donation experience. The intervention group was divided into 2 stages: those with an intervention at 3 months and those with a follow-up 3 months later. After 6 months, the redonation rate was evaluated for the 2 groups, and the scale in the intervention group was determined both before and after the intervention. A *t* test, chi-square test, logistic stepwise regression, and ANOVA were performed.

**Results:**

The intervention group’s redonation rate was 16.14%, which was significantly higher than the control group’s redonation rate of 5.16%; *P*<.001. Men who were aged 31 to 45 years and had donated blood twice had a higher redonation rate after the web-based video intervention than after the SMS text messages; *P*<.05. The repeat donors’ improved blood donation anxiety (*P*=.01), outcome expectations (*P*=.008), and cognition of the blood donation environment (*P*=.005) after the intervention were significantly higher than those of the nonrepeat donors.

**Conclusions:**

The web-based short video intervention based on the extended theory of planned behavior can effectively improve redonation rates. Outcome expectations, blood donation anxiety, and cognition of the blood donation environment can directly influence irregular blood donors to redonate blood.

## Introduction

### Background

Although blood is an indispensable and important resource for clinical treatment, an imbalance between supply and demand may occur as the population ages and diversifies [1]. Studies indicate that repeat blood donors are safe blood sources because of their voluntary blood donation education and frequent blood screening [2-4]. However, the high rate of reduction in the number of first-time voluntary blood donors and the low rate of repeated blood donation among are common problems worldwide. The rate of repeated blood donation in Zhejiang province from 2006 to 2015 was 30.8%, which was lower than the global rate of 50% and fell within the average range of 24.3% to 38.8% in China [5]. Hence, a common challenge—in Zhejiang province as well as nationally and internationally—involves the question of how the rate of repeat blood donations can be increased while ensuring an ample blood supply and safety. Current research on the evaluation, prediction, and behavioral intervention of repeated blood donation behavior is in its infancy. Furthermore, the relationship between personal, psychological, and socioenvironmental factors, among others, and repeated blood donation behavior has been clarified, nor has an authoritative evaluation system been developed to index repeated blood donation intentions [6-8]. The literature has primarily focused on changing blood donation knowledge, attitude, and willingness through education [9,10]. Most methods involve traditional SMS text messages, phone calls, and brochures [11] and lack robustness in methodological reporting [12] and intervention studies on repeated blood donation behavior. Few studies have addressed prospective randomized controlled trials of repeated blood donation intervention.

### Objectives

The theory of planned behavior is the most widely used theory to explain behavioral motivation and has consistently demonstrated the ability to predict blood donation intention and behavior [13-17]. This theory posits that human behavior is determined by 3 aspects: the first factor is the consequences of a behavior and the evaluation of these results, which can generate positive or negative attitudes toward the behavior. The second factor comes from the normative expectations of others and the motivation to follow these expectations, namely normative beliefs, which lead to social pressure and subjective norms. The resources and opportunities required for this behavior, as well as their ease of access, are the control beliefs that lead to the third factor, that is, perceived behavioral control. Although a majority of studies have confirmed that the theory of planned behavior can effectively predict behavioral intentions and can significantly improve the explanatory and predictive power of behavioral research, such works also have various shortcomings, such as the omission of socioenvironmental factors and insignificant intervention effects [18,19]. Ajzen [20] observed that if a factor was found to enhance the prediction of an intention or behavior, the theory of planned behavior can extend the factor, forming an extended theory of planned behavior (ETPB). Therefore, this study’s initial stage first considers a literature review and a Delphi expert consultation based on the theory of planned behavior’s 4 dimensions: attitude, subjective behavioral norms, perceived behavioral control, and willingness. It also explores the expected outcome, self-identity, and blood donation anxiety and environment and ultimately forms an ETPB; further research is incorporated to form a repeat blood donation intention–assessment scale with this theory as the overall guiding framework (Textbox 1).

The “Statistical Report on Internet Development in China” indicates that as of December 2020, China’s short videos reached an audience of 873 million people or 88.3% of all netizens [21]. The widespread popularity of these short videos suggests that people generally accept and enjoy them. Currently, videos are widely used in behavioral health interventions, such as patient education for different diseases and patient family care [22-28], but few studies have examined their application in blood donation environments. On the basis of the previous research results on the factors influencing repeated blood donation as guided by the ETPB [28-30], this study designed short videos based on the ETPB; these short videos were presented on the web to nonregular blood donors as repeated blood donation interventions. An exploratory, prospective, randomized, and controlled experiment was conducted to analyze the changes in intermediary variables before and after the intervention period. The results from repeated blood donation behavior were compared with those from the SMS control group to not only analyze the intervention effect but also provide a reference for empirical research in determining the next intervention strategy to ensure repeat blood donation behavior.

**The influencing factor scale of repeated blood donation based on the extended theory of planned behavior.**

**Factors and the corresponding items**

1. Attitude
  - I think donating blood can save lives.
  - I think donating blood is a kind of blood storage protection for me and my family.
  - I feel that giving blood demonstrates my courage.
  - I think many people in the hospital need blood transfusions and need me to donate blood.
2. Subjective behavioral norms
  - Most of the people who are important to me think I should donate blood or donate again.
  - Most of the people who are important to me will support and encourage me to donate again.
  - Most people I know will evaluate me based on whether I donate blood or donate again.
  - I think donating blood is about everyone.
3. Perceived behavioral control
  - The standardized process of voluntary blood donation will not be infected with diseases.
  - I will pay attention to information on voluntary blood donations (such as those presented on the television, internet, newspapers, or magazines) and will actively acquire knowledge about voluntary blood donation.
  - Each voluntary blood donation of 200-400 mL is in the normal range and will not damage the body.
  - I will take the initiative to donate blood because my family, friends, or colleagues donate blood.
  - I will encourage my family, friends, or colleagues to voluntarily donate blood.
  - It is my decision to donate blood or continue to donate blood again.
  - I can meet the necessary conditions, such as good health or a convenient time, among others, to increase the number of blood donations.
  - If the blood donation experience will be positive, I will donate blood or donate blood again.
  - If my family can prioritize transfusions as necessary after I donate blood, I will donate blood or donate blood again.
  - I am confident I will overcome the factors that may prevent me from donating or continuing to donate blood.
  - The preferential blood donation policy affirmed and encouraged me.
  - in the next year, I plan to donate blood (or donate again).
4. Blood donation willingness
  - I believe that I will be able to donate blood or donate blood again within the next year.
  - Blood donation souvenirs or awards will motivate me to donate blood or donate blood again.
  - In the next year, I will definitely donate blood (or donate again).
5. Outcome expectations
  - If I donate blood again, more patients will be treated.
  - If I donate blood again, I can set a good example for others.
  - If I donate blood again, I will gain more recognition and respect.
  - Voluntarily donating blood at regular intervals (6 months or more) is good for your health.
  - If you do not donate blood, or do not continue to donate blood, you are likely to regret it in the future.
6. Self-identity
  - I am the type of person who will donate blood (or continue to donate blood).
  - I believe it is appropriate in every way for someone like me to donate blood (or donate blood again).
  - Donating blood is a way of realizing one’s self-worth.
7. Donation anxiety
  - I am concerned that my physical condition does not meet the blood donation requirements.
  - Dissatisfaction with the blood donation experience, whether when I have donated blood or heard from others, causes me to worry about donating blood.
  - If I am asked to donate blood or donate blood again, I will feel distressed and anxious.
8. Cognition of the blood donation environment
  - The blood donation environment looks clean and comfortable.
  - The blood donation environment looks safe.
  - The blood collection staff at the donation site are highly skilled.
  - The blood donation site’s hours of operation are convenient for me.
  - The blood donation site’s staff were friendly.
  - The blood donation site’s location was convenient for me.
  - I have seen promotional materials for blood donation in the media.
9. Previous blood donation experience
  - Have you ever felt unbearable pain when donating blood?
  - Have you ever experienced dizziness, weakness, or a mild headache during or after donating blood?
  - Have you ever felt nervous when donating blood?

## Methods

### Research Design

This was a prospective, single-blind, randomized study. SMS text messages from the Zhejiang provincial blood management information system were sent to eligible, nonregular blood donors, inviting them to participate in the study. The text messages included an invitation letter and a research link. Blood donors who were willing to participate could click the link to obtain detailed information, such as the research objective and content, notice of informed consent, and the research group’s contact information. Participants were randomly assigned to either a web-based intervention group or a SMS control group. As blood donors in China have a minimum interval of 6 months between donations, the study’s SMS control group received a regular reminder SMS within the 6-month interval. The web-based intervention group was analyzed across 2 phases: the intervention period, or the first 3 months, and the follow-up period, or the next 3 months. A baseline survey was conducted using the scale before the intervention and reassessed using the same scale at the end of the intervention period. This scale’s outcome measures were the 9 ETPB factors: attitude, subjective behavioral norms, perceived behavioral control, willingness, outcome expectations, self-identity, blood donation anxiety, the blood donation environment, and previous blood donation experience. At the end of the 3-month follow-up period, blood donation results of the intervention and control groups were tracked using the Zhejiang provincial blood management information system. We hypothesized that the blood donors who received the web-based intervention would donate again more often than those in the SMS control group, as mediated by increases in the 9 ETPB factors. The study protocol was approved by the ethics review committee of the Zhejiang provincial blood center.

### Study Participants, Exclusion and Inclusion Criteria, and the Recruitment Method

According to the World Health Organization’s definition, regular blood donors are those who donated blood >3 times and at least once in recent year [31]. This study examines nonregular blood donors; for the convenience of observation, the inclusion criteria were blood donors aged 18 to 55 years, with current physical conditions meeting the requirements for blood donation, and who meet at least one of the following conditions: (1) donated whole blood in 2019 and did not donate again in 2020, consistent with the category of “*lost donor*” or “*those who donated blood at least once in the past 24 months but did not donate blood in the past 12 months*” [32]; or (2) whole-blood donors with fewer than 3 blood donations and who have not donated blood in the last 6 months, including first-time blood donors who had not donated blood in the past. Respondents were excluded if (1) their current physical condition did not meet the blood donation requirements and (2) they were “regular” blood donors or had donated blood at least 3 times and at least once in recent year.

According to the Zhejiang province’s blood donation statistics in 2017 based on the Zhejiang blood information system, approximately 5% of blood donors had repeatedly donated blood within 6 months after meeting the blood donation interval requirement in Zhejiang province, or specifically, the control group’s repeat blood donation rate was 5%. This study assumes that the intervention could consequently increase the repeat blood donation rate by at least 10% [33]. To detect a minimum 10% difference between the control and intervention groups, the repeat blood donation rate of the latter group was expected to be 15%. The target sample size of each group was calculated to be 141 (α=.05, β=.8). The estimated loss to follow-up rate was 25%; thus, the minimum sample size of each group was 176.

**Figure 1:**
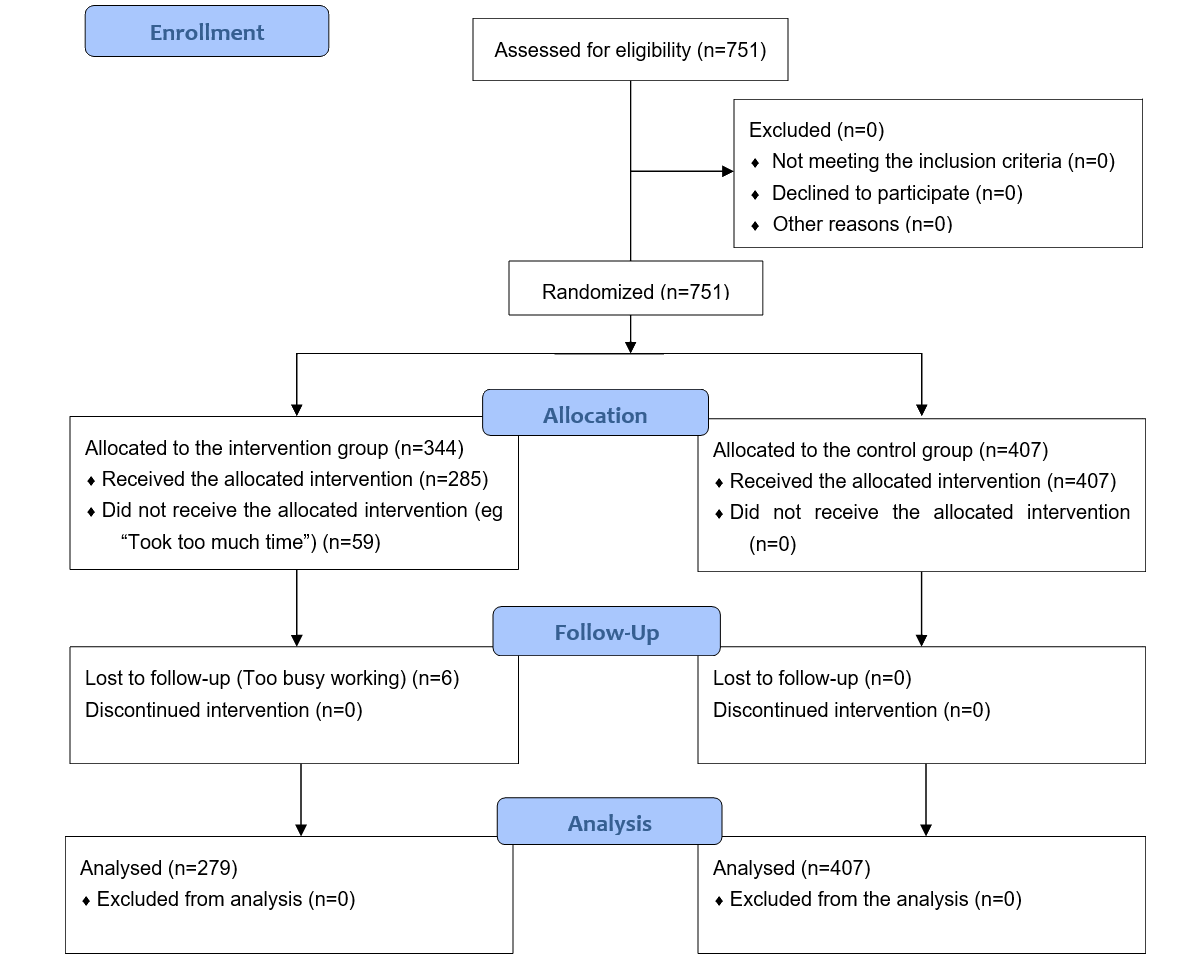
Flow diagram.

From March 9 to 15, 2021, an invitation was texted to all the research participants who met the inclusion criteria. All the 751 respondents who agreed to the invitation were coded by a computer and randomly divided into the web-based intervention (344 people) and SMS control groups (407 people; Figure 1).

### Measurement

#### Web-Based Intervention Methods

Two outcome indicators were used for the web-based intervention: (1) the 3-month period from March 15 to June 15, 2021, was the intervention period, and the changes in the 9 influencing factors before and after the intervention were measured using the same scale and (2) from June 15 to September 15, 2021, the 3-month follow-up period after the intervention period ended, included an investigation of whether the participants donated blood again.

#### Baseline Measurement and Postintervention Reassessment

On the basis of the previous research results, this study adopted the “Repeated Blood Donation Influencing Factors Scale Based on ETPB” (or the “ETPB scale” hereafter) [30], which consists of 9 factors and 44 items. The responses were measured on a 5-point Likert scale and ranged from “strongly disagree” to “strongly agree.” On March 15, 2021, the ETPB scale was sent to the web-based intervention group to collect the participants’ baseline data. On June 15, the day the intervention ended, the same scale was sent again to measure the postintervention results.

### Short Videos Based on the ETPB Elements and Short Videos Regularly Sent on the Web

The primary web-based intervention method involved sending weekly short videos, which were designed based on the ETPB elements, and timely web-based responses to questions from blood donors. This study used smartphones as the carrier because they are characterized as convenient, low cost, and unlimited by time and space, with positive effects, strong communication ability, and high acceptance [34-37]. Moreover, WeChat was chosen because it is easy to operate and free to use and because China’s mainstream social media platform is the most widely used instant messaging tool [38,39]. This study’s web-based intervention WeChat group was equivalent to a small internet-based community. The respondents could view this study’s videos in real time, which facilitates the reception and reading of information and reduces disturbances to daily life while being highly interactive. The group could publicly respond to various frequently asked questions, such as those regarding blood donation locations and policies and how long after vaccination one can donate blood, and eliminate similar doubts among other blood donors.

This study adopted group announcements, real-time communication, and group agency methods in WeChat groups after sending videos. The respondents click a button to complete a group chat after watching a video to let the researchers know that they have watched it. The short videos used in this study were between 45 seconds and 2 minutes in length. Studies have indicated that periodic reminders can encourage the occurrence and persistence of healthy patient behaviors [40-42]. Table 1 displays this study’s short video content and the arrangement of the web-based intervention group.

**Table 1:** Web-based intervention videos’ content and distribution schedule.

Intervention factor	Corresponding short video content	Description	Implementation date
Attitude	1. Why donate blood?	Letting blood donors understand the practical significance of donating blood to save others and promoting change in blood donation attitudes, from opposition and indifference to approval and understanding	Week 1
Self-identity	1. College students’ blood donation stories 2. The blood donor family’s donation story	Self-identity is an important part of self-awareness and the core self-regulatory system in self-awareness. In the human social environment, the process of becoming a qualified social member is inseparable from the growing maturity of self-awareness. This study uses college students’ blood donation and family blood donation experiences to stimulate blood donors’ self-identity regarding blood donation	Week 2
Cognition of blood donation environment	1. The blood donation environment (such as the most beautiful and digital blood donation site)	Sending a video depicting the most esthetic, state-of-the-art blood donation environment to convey the concepts of safety, hygiene, cleanliness, warmth, and convenience	Week 3
Blood donation anxiety	1. Responses regarding blood donation misconceptions 2. Why is there a charge for donating blood? 3. Blood donation knowledge	In providing relief to potential donors by eliminating misunderstandings, this study adopts a face-to-face attitude, with open and candid communication and response methods to reduce or alleviate the blood donors’ anxiety	Week 4
Subjective behavioral norms	1. Stories of regular blood donor representatives 2. Volunteer service	Addressing blood donors’ perceived normative expectations set by others and their motivation to follow those expectations; the video demonstrates that donating blood, as a part of service and selfless dedication to others, can bring spiritual satisfaction and joy and relieve the external pressure that blood donors experience	Weeks 5 and 6
Outcome expectations	1. Blood donation care policy 2. Blood donors get direct fee waived after transfusion 3. One blood recipient’s college car accident story and a Rh-negative recipient’s story	Sending videos of real cases where blood recipients have had their lives saved because of blood transfusions and communicating that timely blood transfusions can avoid the negative consequence of patient death; furthermore, post–blood donation results can include social honors and other care policies that can be enjoyed after donation	Weeks 7 and 8
Previous blood donation experience	1. Precautions taken for donating blood 2. The donation process 3. From one blood vessel to another, 3 topics: the blood source, detection, and blood preparation and supply	Sending videos to reshape the blood donors’ scientific concept of blood donation and view such experiences as adverse reactions in previous blood donation experiences from a scientific perspective	Weeks 9 and 10
Perceived behavioral control	1. Reach out to donate blood 2. People who have donated blood many times show up 3. The first blood donation experience	By addressing the blood donor’s awareness of whether they can donate blood again, the video enhanced the blood donor’s confidence in their ability to donate blood again	Week 11
Blood donation willingness	1. Call for blood donations	The final week’s video reinforces the significance of blood donation as conveyed in the discussion of the first factor. This will hopefully spur recipients to action and change blood donors’ awareness and influence them to donate blood again	Week 12

### Method for the SMS Control Group

In the SMS control group, only regular interval reminder messages were sent during the 6-month period. The content primarily thanked the blood donors for their selfless dedication, warmly reminded them that they have met the minimum required donation interval, and invited them to donate blood again. No other intervention methods were used, such as communication through the telephone or internet. The respondents promised not to watch blood donation–related videos or read similar material during the study.

### Statistical Methods

As SPSS (version 23.0; IBM Corp) software was used to organize the data, the measurement data were expressed as “mean (SD); x [s]),” and the count data were expressed as a percentage (%). Furthermore, this study’s statistical analysis was conducted through a chi-square test, 2 independent sample *t* tests, an ANOVA, and a logistic stepwise regression, among other methods. The results were statistically significant (*P*<.05).

### Ethics Approval

This study was approved by the Regional Ethics Committee of the Blood Center of Zhejiang Province (approval number 2019-019). This study was conducted with the framework of randomized controlled trial, which was in full compliance with the CONSORT guidelines. In terms of content, it has no clinical trials, no human trials, no human samples, no medical records and other information, no human blood samples, pathological phenomena, disease etiology and pathogenesis, no disease prevention, diagnosis, treatment and rehabilitation information, and will not have any adverse effects on the human body. As this study was an observational study, we did not register in the Chinese Clinical Trial Registry. No personal privacy or medical information that can identify the blood donors and commercial interests will be disclosed.

## Results

### Overview

The average age of the participants in this study was 30.47 (SD 9.76) years, approximately 70.4% (487/692) of the participants were male, and the frequency of blood donation was mostly once (481/692, 69.5%) and twice (173/692, 25%). Responses of “*never*” and “*three or more times*” were included only in the intervention group. At the end of 6 months, the intervention group’s blood donation rate was 16.1% and that of the SMS control group was 5.2%. The chi-square test (c^2^_1_=23.1; *P*<.001) results indicated that the difference in repeated donation rates between the groups was statistically significant (Table 2).

**Table 2:** Participants’ demographic and donation information collected during the intervention period^a^.

Items			Web-based intervention group (n=285), n (%)		SMS group (n=407), n (%)
**Sex**					
	Male	198 (69.5)		289 (71)	
	Female	87 (30.5)		118 (29)	
	Intersex	0 (0)		0 (0)	
**Age (years)**					
	18-25	159 (55.8)		98 (24.1)	
	26-30	48 (16.8)		71 (17.4)	
	31-35	27 (9.5)		70 (17.2)	
	36-40	17 (6)		70 (17.2)	
	41-45	14 (4.9)		84 (20.6)	
	46-55	20 (7)		14 (3.4)	
**Number of previous blood donations**					
	0 time	24 (8.4)		0 (0)	
	1 time	130 (45.6)		351 (86.2)	
	2 times	117 (41.1)		56 (13.8)	
	≥3 times	14 (4.9)		0 (0)	
Number of people who donated blood again within 6 months of observation period			46 (16.1)		21 (5.2)

^a^Chi-square test of the blood donation rate for the intervention and control groups during the observation period; c^2^_1_=23.1; *P*<.001.

### Comparative Analysis of the 2 Groups

According to whether the participants in the web-based intervention and SMS control groups donated blood again during the study period, they were divided into the “redonating” and “nonredonating” groups, respectively. The results revealed that male blood donors who were aged 31 to 45 years and had donated twice in the past exhibited significant differences in their response to the text messages and web-based intervention, and the redonation rate was higher among such participants in the web-based intervention group (Table 3).

**Table 3:** Comparative analysis of repeat and nonrepeat donors in the SMS control and web-based intervention groups during the observation period.

Items		SMS group			Web-based intervention group			Chi-square (*df*)		*P* value	
		Repeat donation (n=21), n (%)	Nonrepeat donation (n=386), n (%)	Repeat donation (n=46), n (%)		Nonrepeat donation (n=239), n (%)					
**Sex**											
	Male	13 (62)	276 (71.5)	36 (78)		162 (67.8)	24.3 (1)				<.001
	Female	8 (38)	110 (28.5)	10 (22)		77 (32.2)	1.4 (1)				.24
	Intersex	0 (0)	0 (0)	0 (0)		0 (0)	N/A^a^				N/A
**Age (years)**											
	18-25	5 (24)	80 (20.7)	17 (37)		142 (59.4)	1.6 (1)				.21
	26-30	7 (33)	64 (16.6)	10 (22)		38 (15.9)	2.8 (1)				.09
	31-35	3 (14)	68 (17.6)	6 (13)		21 (8.8)	5.6 (1)				.02
	36-40	1 (5)	67 (17.4)	4 (9)		13 (5.4)	8.3 (1)				.004
	41-45	5 (24)	68 (17.6)	7 (15)		7 (2.9)	14.9 (1)				<.001
	46-55	0 (0)	39 (10.1)	2 (4)		18 (7.5)	1.6 (1)				.22
**Blood donation times**											
	0 time	0	0	0		24 (10.0)	N/A				N/A
	1 time	17 (81)	334 (86.5)	12 (26)		118 (49.4)	3.2 (1)				.07
	2 times	4 (19)	52 (13.5)	30 (65)		87 (36.4)	8.2 (1)				.004
	≥3 times	0 (0)	0 (0)	4 (9)		11 (4.6)	N/A				N/A

^a^N/A: not applicable.

### Results of the Theory of Planned Behavior Scale Comparison in the Web-Based Intervention Group Before and After the Intervention

After the intervention and verification of the respondents’ information, it was determined that 279 people completed both the baseline and postintervention surveys. The statistical results presented in Table 4 indicate the clear effects of the web-based intervention. The 9 factors—specifically, participants’ attitude, subjective behavioral norms, perceived behavioral control, blood donation willingness, expectation of the results, self-identity, blood donation anxiety, cognition of the blood donation environment, and previous blood donation experience—were significantly improved.

**Table 4:** Comparison of the survey results of the theory of planned behavior scale before and after intervention in the web-based intervention group.

Factor	Before, mean (SD)	After, mean (SD)	*t* test (*df*)	*P* value	Improved, mean (SD)	95% CI
Attitude	17.36 (2.338)	18.19 (2.234)	4.770 (278)	<.001	0.84 (2.292)	0.49-1.18
Subjective behavioral norms	14.9 (2.898)	16.22 (2.974)	5.940 (278)	<.001	1.32 (2.91)	0.88-1.76
Perceived behavioral control	51.82 (5.772)	54.44 (6.165)	5.858 (278)	<.001	2.62 (5.848)	1.74-3.50
Blood donation willingness	20.79 (3.114)	22.04 (3.168)	5.697 (278)	<.001	1.25 (2.859)	0.81-1.68
Outcome expectations	12.99 (1.71)	13.66 (1.535)	5.732 (278)	<.001	0.66 (1.507)	0.43-0.89
Self-identity	16.2 (3.274)	17.55 (6.138)	2.755 (278)	.007	1.35 (6.348)	0.38-2.31
Blood donation anxiety	12.46 (1.941)	13.27 (1.802)	6.234 (278)	<.001	0.82 (1.717)	0.56-1.08
Cognition of the blood donation environment	27.25 (4.464)	29.82 (4.138)	9.128 (278)	<.001	2.57 (3.686)	2.02-3.13
Previous blood donation experience	10.82 (2.646)	11.25 (2.855)	2.613 (278)	.01	0.22 (2.745)	0.2-0.63
Total	184.56 (21.124)	196.19 (21.986)	8.931 (278)	<.001	11.64 (17.039)	9.07-14.21

### Comparison of Variables Before and After the Intervention for Blood Donors With Different Blood Donation Times in the Web-Based Intervention Group

The respondents in the web-based intervention group were further divided into groups based on the number of times they had donated blood in the past: none, once, twice, or ≥3 times. Statistically significant differences were observed between the groups in the factors blood donation anxiety, cognition of the blood donation environment, and previous blood donation experience. The willingness to donate, blood donation anxiety, and cognition of the blood donation environment improved the most in the group with 2 donations, and the difference between the groups was statistically significant. In terms of cognitive improvement regarding the respondents’ past blood donation experiences, the group with 1 donation showed greater improvement than the other groups, with a statistically significant difference between the groups (Table 5).

**Table 5:** Comparison of variable changes in blood donors with different blood donation times after the web-based intervention.

Factor	0 time, mean (SD)	1 time, mean (SD)	2 times, mean (SD)	≥3 times, mean (SD)	*F* test (*df*)	*P* value
Attitude	0.63 (2.018)	0.69 (2.303)	0.98 (2.201)	−0.40 (3.376)	1.673 (282)	.17
Subjective behavioral norms	1.21 (3.464)	1.04 (3.002)	1.63 (2.705)	0.20 (4.873)	1.358 (282)	.26
Perceived behavioral control	3.17 (5.239)	2.46 (5.203)	3.04 (4.800)	−1.07 (11.835)	2.484 (282)	.06
Blood donation willingness	0.33 (3.046)	0.89 (3.148)	1.57 (2.595)	−0.40 (4.469)	2.845 (282)	.04
Outcome expectations	0.42 (1.613)	0.45 (1.576)	0.71 (1.527)	0.27 (1.668)	0.822 (282)	.48
Self-identity	−0.67 (2.729)	0.00 (4.334)	0.01 (3.121)	0.40 (2.501)	0.315 (282)	.82
Blood donation anxiety	−0.46 (2.126)	0.66 (1.749)	1.00 (1.698)	−0.13 (1.685)	5.637 (282)	.001
Cognition of the blood donation environment	1.25 (3.904)	1.17 (3.657)	3.16 (3.626)	1.73 (4.284)	6.166 (282)	<.001
Previous blood donation experience	−1.54 (3.189)	0.34 (3.087)	−0.17 (2.857)	−0.33 (2.870)	2.796 (282)	.04
Total	4.33 (13.786)	7.7 (18.078)	11.93 (14.852)	0.27 (26.980)	3.210 (282)	.02

### Postintervention Variable Comparison of Repeat and Nonrepeat Donors in the Web-Based Intervention Group

According to whether they donated blood again after the intervention in the subsequent 6-month period, the respondents in the web-based intervention group were divided into 2 groups, and the differences in the changes in the 9 variables were compared and analyzed. Table 6 reveals that the blood donors who chose to donate blood again after the intervention exhibited a greater improvement in the “outcome expectation” and “blood donation anxiety” variables; compared with nonrepeat donors, the difference was statistically significant.

Furthermore, with the blood donation result again as the dependent variable, age, gender, blood donation frequency, and the 9 intermediary variables were included as independent variables. A logistic stepwise regression indicated that improvements to the “outcome expectations” and “blood donation environment” factors can increase the possibility that nonregular blood donors will donate again, with statistical significance (Table 7).

**Table 6:** Analysis of the degree of change in variables among the repeat and nonrepeat donors in the web-based intervention group after the intervention.

Factor	Nonrepeat donors, mean (SD)	Repeat donors, mean (SD)	*t* test (*df*)	*P* value
Attitude	18.19 (2.096)	18.34 (2.854)	0.441 (283)	.66
Subjective behavioral norms	16.36 (2.971)	16.24 (3.226)	−0.25 (283)	.80
Perceived behavioral control	54.19 (5.543)	54.85 (7.430)	0.693 (283)	.49
Blood donation willingness	21.88 (3.184)	22.35 (3.466)	0.889 (283)	.38
Outcome expectations	13.45 (1.679)	14.04 (1.264)	2.739 (283)	.008
Self-identity	15.22 (3.391)	16.11 (3.295)	1.626 (283)	.11
Blood donation anxiety	13.06 (1.898)	13.70 (1.412)	2.603 (283)	.01
Cognition of the blood donation environment	30.18 (4.333)	29.52 (3.650)	−1.082 (283)	.28
Previous blood donation experience	13.32 (3.493)	14.26 (3.022)	1.701 (283)	.09
Total	195.86 (20.573)	199.41 (20.824)	1.068 (283)	.29

**Table 7:** Results of the logistic stepwise regression analysis of the web-based intervention group after the intervention.

	B	SE	Chi-square (df)	*P* value	Exp (B)	95% CI
Outcome expectations	0.560	0.174	10.3 (1)	.001	1.751	1.244-2.465
Blood donation environment	0.165	0.059	7.8 (1)	.005	0.848	0.754-0.952
Constant	−3.886	1.871	4.3 (1)	.04	0.021	N/A^a^

^a^N/A: not applicable.

## Discussion

### Principal Findings

This study aimed to not only address the possible influencing factors of blood donor losses after an initial blood donation but also discover theoretical intervention strategies given these factors to improve repeat donation rates. This study first provided data on interventions for nonregular blood donors based on the ETPB, which was important in providing reasons for continuous blood donation and future intervention directions; the results then indicated the effects of 2 different intervention methods: web-based videos and SMS text messages.

There are 3 factors that have a significant influence on repeated blood donation behavior: outcome expectations, blood donation anxiety, and blood donation environment. The first step in planning blood donation interventions involves having knowledge regarding the preventive factors in blood donation [43]. This study applied an ETPB to a prospective randomized controlled trial of an intervention in repeat blood donation behavior and received positive results. After the intervention, participants’ attitudes, subjective behavioral norms, perceived behavioral control, blood donation willingness, self-identity, blood donation anxiety, outcome expectations, cognition of the blood donation environment, and cognition of the previous blood donation experience all significantly improved, with positive changes.

However, improvements in such perceptions as attitudes were not always reflected in the respondents’ actions [44-46], and actual blood donors were far fewer than self-reported blood donors. Therefore, what factors have a significant impact on repeated blood donation behavior? This study further observed that 3 factors—outcome expectations, blood donation anxiety, and cognition of the blood donation environment–significantly differed between those who chose to donate blood again after the web-based video intervention and those who did not donate blood again after the intervention. Clearly, these 3 factors significantly impacted repeat blood donation behaviors.

First, outcome expectations can be divided into positive outcome expectations and negative outcome expectations. Similar to other studies, negative outcome expectations, such as anticipatory regret, have been shown to predict blood donation behavior [47-49]. Simultaneously, studies have demonstrated that in promoting healthy behaviors, the persuasion effect to avoid loss will be better than that to obtain gains [50]. This study adopted a negative outcome expectation, and 2 videos of negative outcome expectation were presented. One was about college students in a car accident who required substantial blood transfusions during surgery. The video indicated that if everyone actively donated blood, the blood supply would be sufficient to avoid any negative consequences, including amputations. The other one was about a Rh-negative mother in childbirth who urgently needed a transfusion. If everyone actively donated blood, an adequate supply of blood would ensure a smooth delivery, and the mother would avoid the negative consequences of stillbirth or infant death. These videos aroused donors’ empathy, generated positive emotions, and psychologically matched the act of donating blood with the individual in need of help, prompting people to donate blood again. In support of the suggestion that the transfusion story videos should be promoted more in the future, these videos also helped people realize that repeatedly donating blood could avoid loss of life for the recipients because of insufficient blood supply; the viewers could avoid regret and be encouraged to donate blood again.

Second, this study verified that blood donation anxiety was an important factor affecting repeat donation behaviors; blood donation anxiety can prevent repeat donations. Other studies have shown that blood donation anxiety was critical in blood donors’ decision to donate blood again [51]. The main reasons for not donating blood were concerns about safety and fear of donation [52,53]. Previous studies have revealed the fear of donating blood, needles in particular, and the belief that blood donation will adversely affect one’s health are primary anxiety factors [54-56]. Hence, this study’s short video of blood donation anxiety factors was aimed at explaining the above major anxiety factors in a straightforward manner and refuting the common fears and misunderstandings in donating blood. The video also details the entire donation process and the practices of blood donation testing, blood donation preparation, and delivery of the donated blood to the hospital, thereby reducing misunderstandings, alleviating blood donors’ fears, and enhancing safety as well as confidence in the blood supply.

Third, the research discovered the connections between these environmental factors and blood donation behavior. A good blood donation environment may promote repeated blood donation behavior. Therefore, the possibility of irregular blood donors’ repeat donations can be increased by improving the blood donation environment and providing a warm and comfortable blood donation environment, mitigating blood donation anxiety and strengthening outcome expectations. This is an important finding in research on repeat blood donors after expanding the theory of planned behavior in this study, and it offers significance and guidance for blood collection and supply institutions in implementing their own interventions for nonregular blood donors in subsequent steps.

In addition, the study also found that men aged 31 to 45 years and had donated blood twice in the past and irregular blood donors who had donated twice in the past were more likely to donate blood again after the web-based intervention than after the SMS text message. This may be related to the fact that those who have donated blood twice have had a certain donation experience and a particular foundation in blood donation knowledge, including the process, experience, and perceptions. The group with ≥3 donations had the most experience in donating blood; with a similar “ceiling effect” [57], there was limited room for cognitive improvement. Therefore, those who had donated twice in the past were the most likely to become regular donors. Blood donors aged over 31 years generally had steady employment and were more mature. After receiving the relevant video interventions, they exhibited a higher action-based conversion rate after a cognitive change.

Web-based video interventions were effective. A major issue for blood donation workers involves the question of how to not only best convey information on coping with the obstacles to blood donation but also choose the best intervention method. This study combines the currently most effective web-based short video methods for dissemination with guidance from an ETPB to conduct an exploratory study of behavioral interventions. The study’s results—specifically, that the web-based short video intervention method was more effective than SMS text messages for nonregular blood donors—were consistent with the research findings that video can effectively improve patients’ knowledge, self-efficacy, satisfaction, and self-management levels in other areas, such as diabetes, heart disease, and patient family care [22-28,58].

However, this study differs from the findings of Karacaoğlu and Öncü [59]. Karacaoğlu and Öncü [59] began with first understanding new blood donors’ fears and concerns and compared 6-minute educational videos with the brochures in use at that time; the videos addressed how to handle stress and anxiety among those experiencing the blood donation procedure for the first time. Considering the increase in knowledge and decrease in anxiety as outcome indicators after the intervention, the results revealed no difference between the brochure and video intervention groups. In the study by Masser et al [11], the video content was relatively simple, with only a video providing content from a precaution manual on the process before, during, and after blood donation. In contrast to these studies, this study first provided a short video with rich content, which was theoretically guided, driven by influencing factors, and provided on the web; measured the degree of psychological change from the intervention; and then tracked blood donation behavior rather than blood donation intention as an outcome variable, which more intuitively reflects the overall situation from the change of consciousness to the occurrence of behavior. The study also found that when those viewing the web-based video intervention chose to donate blood again, most of them uploaded photos of the time when they donated blood again to the WeChat group. This also played a role in donors’ taking the initiative by example and encouraged undecided blood donors to donate blood again.

### Conclusions

In conclusion, this study developed and verified an ETPB-driven web-based video intervention method to promote nonregular blood donors to donate again. The method addressed the individual donors’ psychological and environmental factors, with remarkable results that can be popularized and applied in nonregular blood donor interventions to consequently improve repeat blood donation rates. Among the factors presented in this study, blood donation anxiety, result expectation, and improvements to the blood donation environment can positively impact repeat blood donation behaviors; hence, these are recommended as directions of focus in subsequent key interventions.

However, this study also has some limitations. First, this was an exploratory study, and its video sequence and frequency corresponding to the influencing factors were the first attempt and exploration, with no comparative study of other sequences. Second, the web-based intervention was based on the WeChat social media platform. Although group announcements and tasks were available to urge participants to click and watch the videos, no exact, effective means were used to understand more specific information, such as a particular viewing time. The respondents in the SMS group promised not to watch videos or other blood donation recruitment material during the study period, but these were limited by the respondents’ self-awareness. As we could not discern whether they actually accessed intervention videos, errors may exist in that some respondents could have still accessed such videos. Third, this study used multiple comparisons, which may have caused a type 1 error. Fourth, this study has only been conducted for 6 months, and a longer follow-up period is needed for more comprehensive results. Fifth, the data collected in this study were Chinese, and the results may be different from those of other countries with different regions and cultures.

In subsequent research, we will continue to track these respondents’ repeat donation behaviors after 1 and 1.5 years to further improve this study. Simultaneously, using the 9 variables discovered in this study—especially outcome expectations, blood donation anxiety, and cognition of the blood donation environment—specific improvement measures were designed and applied to a larger number of nonregular blood donors to observe the results of repeat blood donations. Further research will be conducted on the web-based video intervention method driven by the ETPB created in this study and focusing on factors such as video length, playback order, and sending frequency to further improve the web-based video intervention method.

## Appendix

Multimedia Appendix 1 CONSORT-eHEALTH checklist (V 1.6.1).

## Abbreviations

ETPB: extended theory of planned behavior
